# Artificial intelligence for radiological paediatric fracture assessment: a systematic review

**DOI:** 10.1186/s13244-022-01234-3

**Published:** 2022-06-03

**Authors:** Susan C. Shelmerdine, Richard D. White, Hantao Liu, Owen J. Arthurs, Neil J. Sebire

**Affiliations:** 1 Department of Clinical Radiology, Great Ormond Street Hospital for Children, London, UK; 2 Great Ormond Street Hospital for Children, UCL Great Ormond Street Institute of Child Health, London, UK; 3 Great Ormond Street Hospital NIHR Biomedical Research Centre, London, UK; 4 Department of Clinical Radiology, St. George’s Hospital, London, UK; 5 Department of Radiology, University Hospital of Wales, Cardiff, UK; 6 School of Computer Science and Informatics, Cardiff University, Cardiff, UK

**Keywords:** Artificial intelligence, Machine learning, Fracture, Trauma, Diagnostic accuracy

## Abstract

**Background:**

Majority of research and commercial efforts have focussed on use of artificial intelligence (AI) for fracture detection in adults, despite the greater long-term clinical and medicolegal implications of missed fractures in children. The objective of this study was to assess the available literature regarding diagnostic performance of AI tools for paediatric fracture assessment on imaging, and where available, how this compares with the performance of human readers.

**Materials and methods:**

MEDLINE, Embase and Cochrane Library databases were queried for studies published between 1 January 2011 and 2021 using terms related to ‘fracture’, ‘artificial intelligence’, ‘imaging’ and ‘children’. Risk of bias was assessed using a modified QUADAS-2 tool. Descriptive statistics for diagnostic accuracies were collated.

**Results:**

Nine eligible articles from 362 publications were included, with most (8/9) evaluating fracture detection on radiographs, with the elbow being the most common body part. Nearly all articles used data derived from a single institution, and used deep learning methodology with only a few (2/9) performing external validation. Accuracy rates generated by AI ranged from 88.8 to 97.9%. In two of the three articles where AI performance was compared to human readers, sensitivity rates for AI were marginally higher, but this was not statistically significant.

**Conclusions:**

Wide heterogeneity in the literature with limited information on algorithm performance on external datasets makes it difficult to understand how such tools may generalise to a wider paediatric population. Further research using a multicentric dataset with real-world evaluation would help to better understand the impact of these tools.

**Supplementary Information:**

The online version contains supplementary material available at 10.1186/s13244-022-01234-3.

## Key points

- Most artificial intelligence tools for fracture detection on children have focussed on plain radiographic assessment.
- Almost all eligible articles used training, validation and test datasets derived from a single institution.
- Strict inclusion and exclusion criteria for algorithm development may limit the generalisability of AI tools in children.
- AI performance was marginally higher than human readers, but not significantly significant.
- Opportunities exist for developing AI tools for very young children (< 2 years old), those with inherited bone disorders and in certain clinical scenarios (e.g. suspected physical abuse).

## Background

It is estimated that up to a half of all children sustain a fracture at some point during childhood [1, 2] (~ 133.1 per 10,000 per annum). Fractures also represent a leading cause for long-term disability in children [3] and are present in 55% of children who have been physically abused [4]. Given the differences in children’s bone appearances on imaging compared to adults (including differences at varying stages of bone maturation), and the different patterns of injury (such as buckle/torus fractures, corner metaphyseal injuries, bowing deformities), emergency physicians, who are the frequently the first to review and act upon imaging findings, can miss up to 11% of acute paediatric fractures, compared to a specialist paediatric radiologist [5–8]. Of these, the majority (7.8%) could lead to adverse events and changes in management [8]. This is particularly concerning given that over half (57%) of all UK paediatric orthopaedic-related litigation cases relate to undetected or incorrectly diagnosed injuries, costing £3.5 million, with an average pay-out of between £28,000 and £57,000 per case [9, 10]. These results are not limited to UK practice, with similar results from Norway [11] and the USA [12, 13], where paediatric claims resulted in higher indemnity paid per case compared with adults [12, 14].

One potential solution would be the use of artificial intelligence (AI) algorithms to rapidly and accurately abnormalities, such as fractures, on medical imaging. Such algorithms could be useful as an interpretative adjunct where specialist opinions are not always available. A systematic review of AI accuracy for adult long bone fracture detection on imaging reported pooled sensitivity and specificity rates of 96 and 94%, respectively [15]. Another systematic review [16] reported that several AI algorithms [17–21] were either as good or better at detecting limb fractures on radiography compared to general physicians and orthopaedic surgeons. Whilst a minority of studies included any paediatric cases within their training dataset for algorithm development [22, 23], few have analysed how well these perform specifically and solely for the paediatric population.

The objectives of this systematic review are to assess the available literature regarding diagnostic performance of AI tools for paediatric fracture assessment on imaging, and where available, how this compares with the performance of human readers.

## Materials and methods

Ethical approval was not required for this retrospective review of published data. This study was registered in PROSPERO International prospective register of systematic reviews, CRD42020197279 [24]. The updated PRISMA (Preferred Reporting Items for Systematic reviews and Meta-Analyses) statement guidelines were followed [25] (Additional file 1)**.**

### Literature review

MEDLINE (Ovid), EMBASE, Web of Science and the Cochrane Library databases were searched for eligible articles published between 1 January 2011 and 31 December 2021 (11 years range), using database specific Boolean search strategies with terms and word variations relating to ‘fracture’, ‘artificial intelligence’, ‘imaging’ and ‘children’. The full search strategy was conducted on 1 January 2022 (Additional file 1: Tables S1–S4). A repeat search was conducted on 18 February 2022 and again on 30th April 2022 to assess for interim publications since the original search.

### Eligibility criteria

Inclusion criteria encompassed any work investigating the diagnostic accuracy for classification, prediction or detection of appendicular fractures on any radiological modality in children, using one or more automated or artificial intelligence models. Expert radiological opinion, follow-up imaging or surgical/histopathological findings were all considered acceptable reference standards. Studies were limited to human subjects aged 0–20 years, to include adolescents. No restrictions were placed on method of imaging, dataset size, machine vendor, type of artificial intelligence/computer-aided methodology or clinical setting.

Exclusion criteria included conference abstracts, case reports, editorials, opinion articles, pictorial reviews and multimedia files (online videos, podcasts). Articles without a clear reference standard, clear subgroup reporting (to assess whether a paediatric cohort was analysed) or those relating to robotics or natural language processing (NLP) rather than image analysis were excluded. We excluded any animal studies and those referring to excised bone specimens.

All articles were independently searched by two reviewers (both paediatric radiologists with prior experience of conducting systematic reviews and meta-analyses). Abstracts of suitable studies were examined, and full papers were obtained. References from the retrieved full text articles were manually examined for other possible publications. Disagreements were resolved by consensus.

### Methodological quality

Given the lack of quality assessment tools specifically designed for artificial intelligence methodology [26], we used the modified Quality Assessment of Diagnostic Accuracy Studies (QUADAS-2) criteria [27] with consideration of several items outlined from the Checklist for Artificial Intelligence in Medical Imaging (CLAIM) guideline [28].

These are as follows:

1. Patient Selection, risk of bias: consideration regarding appropriate patient selection for the intended task, collating a balanced data set, suitable data sources, unreasonable/extensive exclusion criteria
2. Patient Selection, applicability: how applicable/useful the algorithm for intended usage, given the patient selection.
3. Index test, risk of bias: consideration of measures of significance and uncertainty in the test;
4. Index test, applicability: information on validation or testing of the algorithm on external data;
5. Reference Standard, risk of bias: sufficient detail to allow replication of ground truth/reference standard, whether reader was blinded to clinical details;
6. Reference Standard, applicability: appropriateness for clinical practice.

This combined assessment using QUADAS-2 and CLAIM has been previously employed by other authors for systematic reviews evaluating artificial intelligence studies [29]. Due to the low number of studies fulfilling our inclusion criteria, it was decided a priori to not exclude any studies on the basis of quality assessment to allow as complete a review of the available literature possible.

### Data extraction and quantitative data synthesis

Two reviewers independently extracted data from the full articles into a database (Excel, Microsoft, Redmond WA, USA). A descriptive approach was used to synthesise the extracted data. Information regarding the datasets in terms of the number of images, types of images, and number of diagnostic classes within the data set was collected and recorded. The evaluation metrics (i.e. diagnostic accuracy rates) used in each dataset for each study were described. Due to the heterogeneity of data and body parts assessed, it was planned a priori to provide a narrative description of the results.

## Results

### Eligible studies

**Fig. 1:**
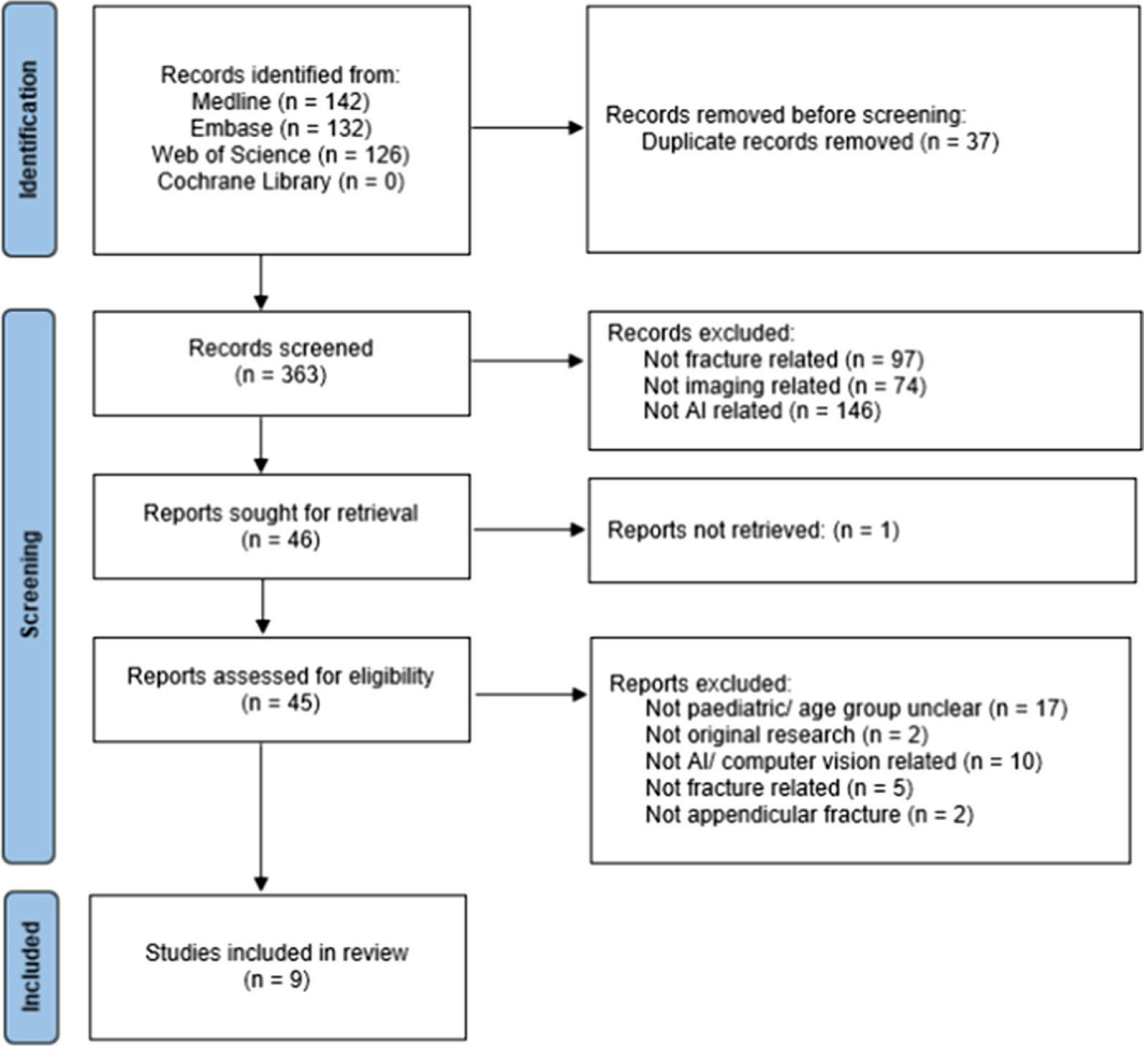
PRISMA flow chart for the study search and selection

The initial search performed on 1 January 2022 yielded 362 articles, after the removal of duplicate studies. On the basis of study title and abstract, 318 articles were excluded or irretrievable. After review of the full text (*n* = 44), eight studies were eventually included [17, 30–36]. An additional search of the medical literature on 18 February 2022 revealed one additional study. A PRISMA flowchart is shown in Fig. 1.

### Methodological quality assessment

**Fig. 2:**
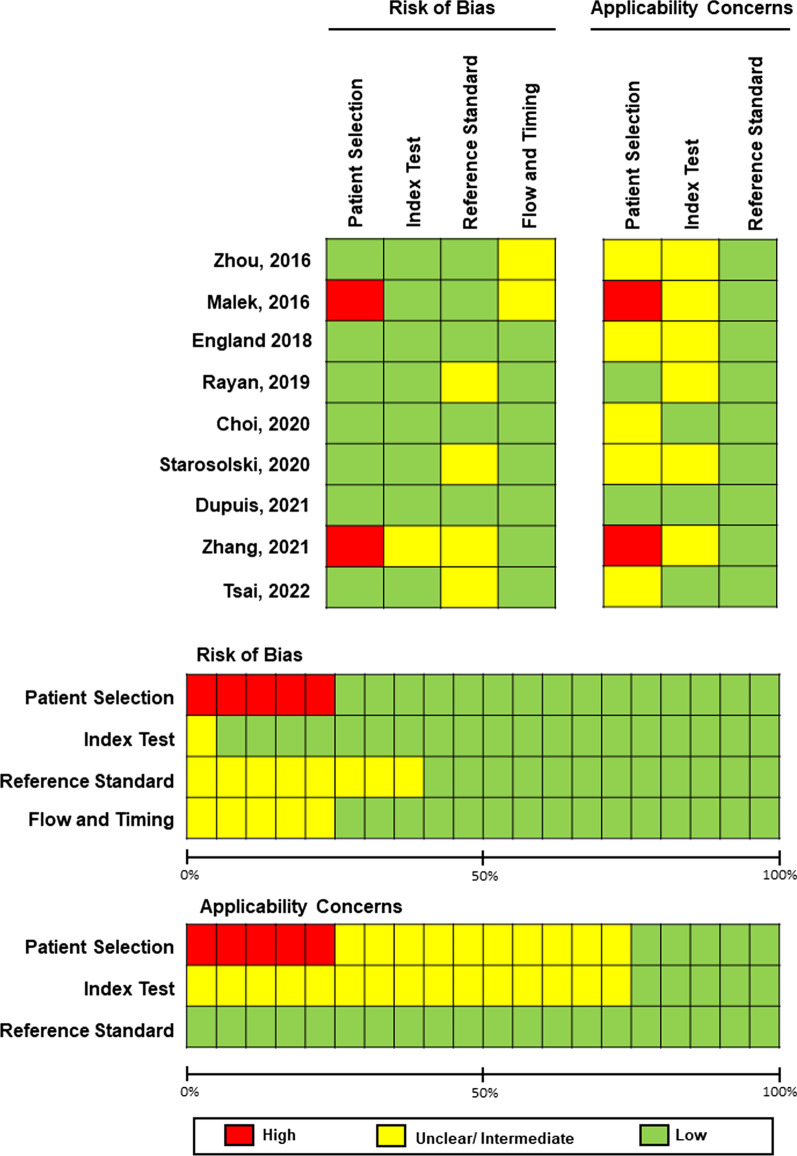
Methodological quality assessment of the included studies using the QUADAS-2 tool. Risk of bias and applicability concerns summary about each domain are shown for each included study

The risk of bias and applicability of the various studies are outlined in Fig. 2. In two studies, there was a high risk of bias and applicability concerns regarding patient selection [32, 35]. In one of these [35], a 3-dimensional ultrasound sweep of the distal radius was performed by medical students on a ‘convenient sample’ of children attending the emergency department with wrist injuries. Patients were neither consecutive, nor randomly sampled; therefore, it was questionable as to how generalisable the study results could be. In the second study [32], children were only included if they had a confirmed lower limb fracture, and were labelled as having either normal fracture healing time or delayed fracture healing (> 12 weeks). The mechanism for follow-up to determine fracture healing time, or the reason for choosing a 12-week time frame, was not specified, and furthermore it was not stated whether children with pre-existing bone fragility disorders were included.

Almost half of all studies had unclear/moderate concerns regarding applicability of patient selection (4/9, 44.4%) [31, 34, 36, 37], and most had concerns regarding applicability of index test (6/9, 66.7%) [31–36]. This was predominantly due to studies imposing strict exclusion criteria in their patient selection (e.g. exclusion of patients with healing bones, certain types of fractures, and treatment with cast or surgical correction devices) which would limit the application of the algorithm in clinical practice. In four studies the risk of bias for the reference standard was considered unclear/moderate as the radiology readers were unblinded to the clinical history, which may have influenced their reporting of findings and subsequent algorithm performance [33–35]. Only two studies reported results for external validation of their algorithm using a dataset which was distinct to the training and validation datasets [17, 30].

### Patient demographics and study setting

The list of studies included, study aims, and patient inclusion/exclusion criteria are provided in Table 1. Patient demographics, type of centre and ground truth/reference levels are covered in Table 2. The majority of the studies (5/9, 55.6%) involved assessment of paediatric upper limb trauma, with three assessing the elbow and two assessing the forearm. One study assessed any fracture of the appendicular skeleton, and the remaining three assessed trauma of the lower limb.

**Table 1:** Study aims, injury to be detected and patient inclusion/exclusion criteria, organised by publication date

Author, year	Country	Body part	Type of injury	Patient inclusion criteria	Patient exclusion criteria	Study aim
Zhou, [36]	USA	Forearm	Plastic bowing deformities	Forearm radiographs of children aged 1–18 years with history of trauma	None stated	Development of a computer-aided detection application for plastic bowing deformity fractures in paediatric forearms
Malek [32]	Malaysia	Lower limb (femur, tibia, fibula)	Any fracture	Radiographs of fractured femur, tibia or fibula in children < 12 years of age	None stated	Development of an artificial neural network to analyse normal (< 12 weeks) versus delayed healing time for paediatric lower limb fractures
England [31]	USA	Elbow	Traumatic elbow joint effusions	Elbow radiographs of children aged 1–19 years attending the emergency department with history of blunt trauma. Lateral view of radiograph technically adequate	Images with cast applied, elbow dislocation/displacement, comminuted fracture, metallic surgical hardware	Detection of traumatic paediatric elbow joint effusions using a deep convolutional neural network
Rayan [33]	USA	Elbow	Any elbow fracture	Elbow radiographs in children	None stated	Binomial classification of elbow fractures using a deep learning approach
Choi [17]	South Korea	Elbow	Supracondylar fractures	Elbow radiographs (two views) in children with suspected supracondylar fracture	Follow-up imaging (only initial radiographs included) Non-supracondylar fractures Elbow dislocation Underlying bone dysplasia	Development of a dual input convolutional neural network for detection of supracondylar fractures
Starosolski [34]	USA	Distal tibia	Most fracture types	Radiographs of the foot, ankle, tibia or fibula in children	Plastic bowing fractures or any fracture without discrete fracture line. Images with surgical fixation, cast or other alternative pathology than fracture	Development of a convolutional neural network for detection of tibial fractures
Dupuis [30]	France	Appendicular skeleton	Any appendicular fracture type	Radiographs of any body part from consecutive patients < 18 years old with suspected trauma attending emergency department	Radiographs of the axial skeleton (skull, spine, chest)	External validation of a commercially available deep learning algorithm for appendicular fracture detection in children
Zhang [35]	Canada	Distal radius	Any fracture type	Children aged < 17 years with unilateral distal radial tenderness following trauma with asymptomatic contralateral wrist as normal comparator	Existing cast over forearm, laceration of the forearm, open fractures, inability to tolerate ultrasound study, lack of time for scanning	Diagnostic accuracy of 3D ultrasound and use of artificial intelligence for detection of paediatric wrist injuries
Tsai [58]	USA	Distal tibia	Corner metaphyseal fractures	Children aged < 1 years referred for suspected abuse	None stated, AP projections for normal and abnormal distal tibial radiographs included only	Develop and evaluate a machine learning based binary classification algorithm to detect distal tibial corner metaphyseal fractures on radiographic skeletal surveys performed for suspected infant abuse

**Table 2:** Study characteristics for articles included in systematic review, organised by publication date

Author, year	Dataset study period	Patient ages (years, unless otherwise stated)	% Male	No. centres	Type of centre(s)	Index test	Ground truth / reference	Ground truth blinded to clinical detail?
Zhou [36]	Not stated	Range: 1–18	Not stated	Single	Tertiary Paediatric	Plain radiography	Two radiologists, over 10-year experience each	Yes
Malek [32]	4 years (2009–11, 2014)	Median: 8.5 SD: 3.9 Range: 0–12	Not stated	Single	Tertiary Paediatric	Plain radiography	Time to fracture healing where no fracture line can be identified on radiography, as determined by single orthopaedic surgeon	No, but all cases were fractured
England [31]	3.6 years (Jan 2014–Sept 2017)	Mean: 11.4 SD: 5.1 Range: 1–19 Percentage of children in age groups (1–5, 6–10, 11–15, 16–19) per dataset are also provided in manuscript.	64.6%	Single	Tertiary Paediatric	Plain radiography	Radiology reports by consultant radiologist. A sub selection of 262 mages re-reviewed by three musculoskeletal radiologists	Musculoskeletal radiologists assessing a sub selection of the radiographs were blinded. Original radiologist report unblinded
Rayan [33]	4 years (Jan 2014–Dec 2017)	Mean: 7.2 Range: 0–18	57%	Single	Tertiary Paediatric	Plain radiography	Radiological reports by a single radiologist (experience unspecified)	No
Choi [17]	6 years (Jan 2013 to Dec 2018)	Percentage of children in age groups (0–4, 5–9, 10–14, 15–19) per dataset are provided in manuscript. No mention of mean, median ages overall. Range: 0–19	Not stated	Two centres, same city	Tertiary Paediatric	Plain radiography	All radiographs re-reviewed by two paediatric radiologists	Yes
Starosolski [34]	8 years (2009–2017)	Mean: 6.4 SD: 4.4	33%	Single	Tertiary Paediatric	Plain radiography	Radiology reports by a single radiologist	Unclear
Dupuis [30]	1 year (March 2019–2020)	Median: 9.2 Mean: 8.5 Range: 0–17 SD: 4.5	57.3%	Single	Tertiary Paediatric	Plain radiography	Radiology report by one of a possible eleven radiologists with 2.5–35 years’ experience	No, but this reference was not used for training
Zhang [35]	Not stated	Mean: 9.9 Range: 3.8–14.8	70%	Single	Tertiary Paediatric	3D ultrasound	Plain radiography acquired within 30 days of ultrasound of affected wrist, reported by consultant radiologist of affected limb. The contralateral limb was also imaged with ultrasound but without radiography confirmation of injury. In these cases normality was presumed where asymptomatic	Not for the 3D ultrasound, unclear regarding radiography reporting
Tsai [58]	13.4 years (1 Jan 2009 to 31 May 2021)	‘Normal’ Cohort Mean: 5 months Range: 0.2–11.6 months SD: 3.3 months. ‘Abnormal’ Cohort Mean: 3.3 months Range: 0.4–12 months SD: 2.9 months	‘Normal’ Cohort = 68.5%; ‘Abnormal’ Cohort = 73%	Single	Tertiary Paediatric	Plain radiography	Radiology report issued by consultant radiologist with subsequent confirmation by primary study author (experienced paediatric radiologist)	Unclear, likely not blinded

In three of the studies, children below the age of 1 year were not included in the study dataset and in one study the age range was not provided. In three studies, the gender split of the dataset was not reported, and none of the studies provided details regarding the ethnicity or socio-economic class of the patients.

The majority of studies (8/9, 88.9%) used datasets which were derived from the author’s own institution (i.e. a single centre study), and analysed fractures on plain radiography. Only one study reported the development of an AI algorithm for fracture detection using ultrasound. The ground truth/reference level for fracture assessment was from the radiology report (7/9, 77.8%), the opinion of an orthopaedic surgeon (1/9, 11.1%) and in the one study related to ultrasound assessment, the corresponding plain radiography report acquired within 30 days of the ultrasound acted as the reference standard for presence of forearm fracture.

### Imaging dataset sizes

The total datasets within the articles were described in different ways, some in terms of number of patients or number of examinations (where each consisted of multiple images) and some in terms of the total number of images. Datasets ranged from between 30 and 2549 patients; 55–21,456 examinations; and 226–58,817 images. Depending on the aims and objectives of each study, some provided a breakdown of the number of examinations (and the split between normal and abnormal examinations) as well as the number of images allocated to training, validation and testing. Full details are provided in Table 3.

**Table 3:** Input data demographics and study dataset sizes, organised by publication date

Author, year	Body part	Total dataset (patients)	Total dataset (exams and images)	Training set	Validation set	Test set
Zhou [36]	Forearm	226	226 radiographs (59 bowing fractures)	226 radiographs (59 bowing fractures)	N/A	N/A
Malek [32]	Lower limb (femur, tibia, fibula)	57	Unclear, presumed 57 exams. No mention of projections or total images. (25, 50% normal healing time; 25, 50% delayed healing time)	39 exams (18, 50% normal; 18, 50% abnormal)	9 exams (4, 44.4% normal; 5, 55.6% abnormal)	17 exams (11, 64.7% normal; 6, 35.3% abnormal)
England [31]	Elbow	882	901 lateral radiographs (images)	657 images (500, 76.2% normal; 157, 23.8% abnormal)	115 images (82, 71.3% normal; 33, 28.7% abnormal)	129 images (96, 74.4% normal; 33, 25.6% abnormal)
Rayan [33]	Elbow	Not stated	21,456 exams; 58,817 images	20,350 exams; 55,721 images (4966, 24% normal, 15,384, 76% abnormal)	1106 exams; 3096 images (516, 47% normal, 590, 53% abnormal)	N/A
Choi [17]	Elbow	810	1619 elbow exams; 3238 images	1012 exams (780, 77.1% normal; 232, 22.9% abnormal)	254 examinations (196, 77.2% normal; 58, 22.8% abnormal)	Temporal set: 258 exams (192, 74.4% normal; 66, 25.6% abnormal) Geographic set: 96 exams (72, 75.8% normal, 23, 24.2% abnormal)
Starosolski [34]	Distal tibia	490	490 exams; 245, 50% abnormal 245, 50% normal	Not stated	Not stated	98 images (49, 50% normal; 49, 50% abnormal)
Dupuis [30]	Appendicular skeleton	2549	2634 exams; 5865 images	N/A	N/A	1825, 69.2% normal; 809, 30.8% abnormal exams
Zhang [35]	Distal radius	30	55 × 3D ultrasound ‘sweeps’ of both wrists (injured and contralateral); Each ‘sweep’ having ~ 382 image slices Overall 19 cases of distal wrist fracture	21 sweeps (~ 6000 images) Abnormal: Normal split not stated	1640 image slices selected from 72 sweeps of 36 patients.23, 64% normal; 13, 36% abnormal cases 990, 60% normal; 650, 40% abnormal images Unclear how this validation dataset was acquired	N/A
Tsai [58]	Distal tibia	124 patients (35 abnormal, 89 normal)	250 radiographs (177 normal, 73 abnormal)	187 radiographs	13 radiographs	50 radiographs

### Imaging algorithm methodology

Technical details regarding methodology and hyperparameters used in the computer-aided/ artificial intelligence algorithm development are summarised in the Additional file 1: Table S5.

In one study, a computer-aided detection (CAD) method was used to generate a graphical user interface (GUI) to automatically extract/segment forearm bones on an image, analyse the curvature and determine presence of underlying bowing/buckling fractures [36]. In another study, a commercially available AI product utilising a deep convolutional neural network (Rayvolve®) [30] was employed. The remainder either developed or re-trained existing convolutional neural networks. One study evaluated the use of self-organising maps (SOM) and also convolutional neural networks in the evaluation of fracture healing [32].

In terms of neural network architecture, the commercially available product (Rayvolve®) was based on a RetinaNet architecture [30], two studies based their neural network on the Xception architecture [33, 34] and one study used the ResNet-50 architecture [17]. For the remainder, the neural network architecture was not described in the study.

### Algorithm diagnostic accuracy rates

The diagnostic accuracy rates for each study are listed according to body part and also data set (e.g. validation or test set) in Table 4. For the most common paediatric body part assessed (elbow), the algorithms tested on the test dataset achieved sensitivities of 88.9–90.7%, with specificity of 90.9–100%. The only study that evaluated fracture detection rate for the whole appendicular skeleton (across multiple body parts) achieved 92.6% sensitivity and 95.7% specificity [30].

**Table 4:** Diagnostic accuracy of artificial intelligence algorithms for fracture detection, organised by body parts

Author, year	Dataset	Body part	AUC	Accuracy, % (95% CI)	Sensitivity, % (95% CI)	Specificity, % (95% CI)	PPV, % (95% CI)	NPV, % (95% CI)	TP	FP	FN	TN
*Upper limb—elbow*												
England [31]	Validation	Elbow effusions	0.985 (0.966–1.00)	NS	NS	NS	NS	NS	NS	NS	NS	NS
	Test	Elbow effusions	0.943 (0.884–1.00)	0.907 (0.843–0.951)	0.909 (0.788–1.00)	0.906 (0.844–0.958)	NS	NS	87	9	3	30
Rayan [33]	Validation	Elbow fractures	0.947 (0.930–0.960)	0.877 (0.856–0.895)	0.908 (0.882–0.929)	0.841 (0.807–0.870)	0.867 (0.838–0.892)	0.889 (0.858–0.914)	536	82	54	434
Choi [17]	Validation	Supracondylar fractures	0.976 (0.949–0.991)	0.945 (0.910–0.967)	0.948 (0.859–0.982)	0.944 (0.902–0.968)	0.833 (0.726–0.904)	0.984 (0.954–0.995)	55	11	3	185
	Temporal test set	Supracondylar fractures	0.985 (0.962–0.996)	0.904 (0.855–0.938)	0.939 (0.852–0.983)	0.922 (0.874–0.956)	0.805 (0.717–0.871)	0.978 (0.945–0.991)	62	15	4	117
	Geographical test set	Supracondylar fractures	0.992 (0.947–1.000)	0.895 (0.817–0.942)	1.000 (0.852–1.000)	0.861 (0.759–0.931)	0.697 (0.564–0.803)	1.000	23	10	0	62
Dupuis [30]	Test	Elbow fractures (subgroup)	NS	0.888 (0.847–0.919)	0.918 (0.846–0.958)	0.873 (0.819–0.913)	0.781 (0.969–0.847)	0.956 (0.915–0.977)	89	25	8	172
*Upper limb—other*												
Zhou [35]	Test set (best performing for AP ulnar view, using optimal central angle measurement of bone)	Forearm (Bowing fracture)	0.992 (NS)	NS	1.000 (NS)	0.940 (NS)	NS	NS	NS	NS	NS	NS
Zhang [35]	Test set—analysed per patient	Distal radius (ultrasound)	NS	0.92	1.0	0.87	NS	NS	NS	NS	NS	NS
*Lower limb*												
Malek [32]	Training	Lower limb fracture healing	0.8 (NS)	0.821 (0.673–0.910)	0.792 (0.595–0.908)	0.867 (0.621–0.963)	0.905 (0.711–0.973)	0.722 (0.491–0.875)	19	2	5	13
	Validation	Lower limb fracture healing	NS	0.556 (0.267–0.811)	0.600 (0.231–0.882)	0.500 (0.150–0.850)	0.600 (0.231–0.882)	0.500 (0.150–0.850)	3	2	2	2
	Test	Lower limb fracture healing	NS	0.889 (0.565–0.980)	1.000 (0.566–1.000)	0.750 (0.301–0.954)	0.833 (0.436–0.970)	1.000 (0.439–1.000)	5	1	0	3
Starosolski [34]	Test	Distal tibia	0.995 (NS)	0.979 (0.929–0.994)	0.959 (0.863–0.989)	1.000 (0.927–1.000)	1.000 (0.924–1.000)	0.961 (0.868–0.989)	47	0	2	49
Tsai [58]	Test (mean and SD for accuracy across models in fivefold cross-validation)	Distal tibia (corner metaphyseal fracture)	NS	0.93 ± 0.018	0.88 ± 0.05	0.96 ± 0.015	0.89 ± 0.036	0.95 ± 0.023	13	2	2	33
	Test (best performing model)	Distal tibia (corner metaphyseal fracture)	NS	0.960 (0.865–0.989)	0.929 (0.685–0.987)	0.972 (0.858–0.995)	0.929 (0.685–0.987)	0.972 (0.858–0.995)	13	1	1	35
*All appendicular skeleton*												
Dupuis [30]	Test	Appendicular skeleton	NS	0.926 (0.915–0.936)	0.957 (0.940–0.969)	0.912 (0.898–0.925)	0.829 (0.803–0.852)	0.979 (0.971–0.985)	NS	NS	NS	NS

95% confidence intervals are omitted where these are not provided in the publication or calculatable by raw values in the confusion matrix

*AP* anterior–posterior, *NS* not stated. *CI* confidence interval. *AUC* area under the curve, *PPV* positive predictive value, *NPV* negative predictive value, *TP* true positive, *FP* false positive, *FN* false negative, *TN* true negative, *SD* standard deviation

In three studies, the performance of the final AI algorithm was tested against independent human readers on the same dataset [17, 31, 35]. The differences in diagnostic accuracy rates are provided in Table 5. England et al. [31] reported their AI algorithm to have a marginally lower diagnostic accuracy rate than a senior emergency medicine trainee in detecting elbow effusions (diagnostic accuracy 90.7% compared to 91.5%), but a greater sensitivity (90.9% versus 84.8%). Zhang et al. [35] reported their AI algorithm to perform better than a paediatric musculoskeletal radiologist in detecting distal radial fractures on ultrasound (92% diagnostic accuracy versus 89%). Choi et al. [17] examined an AI algorithm for supracondylar fracture detection which achieved a greater sensitivity than the summation score of three consultant radiologists (100% versus 95.7%). When this algorithm used as an adjunctive measure for image interpretation, it was able to demonstrate an improved performance for the lowest performing of the three radiologists, with sensitivity rates improving from 95.7% (radiologist acting alone) to 100% (same radiologist with AI assistance). Despite these slight differences in performance across the studies, there was an overlap in the 95% confidence intervals provided suggesting the changes were not statistically significant.

**Table 5:** Studies comparing artificial intelligence algorithms versus (or combined with) human reader, organised by publication date

Author, year	Human/AI	Accuracy, % (95% CI)	Sensitivity, % (95% CI)	Specificity, % (95% CI)	TP	FP	FN	TN
England [31]	AI	0.907 (0.843–0.951)	0.909 (0.788–1.000)	0.906 (0.844–0.958)	87	9	3	30
	PGY5 emergency medicine trainee (non-radiologist)	0.915 (0.852–0.957)	0.848 (0.681–0.949)	0.938 (0.869–0.977)	90	6	5	28
Choi, [17]	AI (Geographical test set)	0.895 (0.817–0.942)	1.000 (0.852–1.000)	0.861 (0.759–0.931)	23	10	0	62
	Summated score of three radiologists (2–7-year experience) from different institution to test dataset	0.975 (0.950–0.988)	0.957 (0.880–0.985)	0.981 (0.953–0.993)	66	4	3	212
	Lowest performing radiologist alone	NS (AUC 0.977 (0.924–0.997))	0.957 (0.781–0.999)	0.972 (0.903–0.997)	NS	NS	NS	NS
	Lowest performing radiologist with AI assistance	NS (AUC 0.993 (0.949–1.000))	1.000 (0.852–1.000)	0.972 (0.903–0.997)	NS	NS	NS	NS
Zhang [35]	AI (Test set—data undefined)	0.920	1.000	0.870	NS	NS	NS	NS
	Human: paediatric musculoskeletal radiologist	0.89 (0.782–0.949)	1.000 (0.833–1.000)	0.833 (0.681–0.921)	19	6	0	30

95% confidence intervals are omitted where these are not provided in the publication

*NS* not stated. *CI* confidence interval. *AUC* area under the curve, *PPV* positive predictive value, *NPV* negative predictive value, *TP* true positive, *FP* false positive, *FN* false negative, *TN* true negative, *PGY* postgraduate year

## Discussion

Almost all published literature relating to AI assessment for acute appendicular fractures in children is based on radiographic interpretation, with fractures of the upper limb (specifically the elbow) being the most common body part assessed. Nearly all articles used training, validation and testing data derived from a single centre, with few performing external validation. When AI tools were compared to the performance of human readers, the algorithms demonstrated comparable diagnostic accuracy rates and in one study improved/augmented the diagnostic performance of a radiologist.

In this review, we focussed on the assessment of computer-aided/artificial intelligence methods for paediatric appendicular fracture detection, given that these are the most commonly encountered fractures in an otherwise healthy paediatric population (accounting for approximately 70–99% of paediatric fractures [37–39], with less than 5% of fractures affecting the axial skeleton [40–42]). Publications related to the application of computer-aided/AI algorithms for paediatric skull and spine fractures have been described. One developed an AI algorithm for detection of skull fractures in children from plain radiographs [43] (using CT head report as reference standard) and reported high AUC values both on their internal test set (0.922) and external validation set (0.870), with improvements in accuracy of human readers when using AI assistance (compared to without). Whilst demonstrating proof of concept, since most radiology guidelines encourage the use of CT over radiographs for paediatric head trauma [44–46], clinical applicability is limited.

In two articles pertaining to spine fractures [48, 49], the authors applied commercially available, semi-automated software tools designed for adults to a paediatric population for the detection of vertebral fractures on plain radiography or dual-energy X-ray absorptiometry (DEXA). They reported low sensitivity for both software (36 and 26%) not sufficiently reliable for vertebral fracture diagnosis. This finding raises an important general issue regarding the need for adequate validation and testing of AI tools in specific patient populations, in this case children, prior to clinical application to avoid potentially detrimental clinical consequences. This was conducted in the current systematic review for one commercially available product (Rayvolve®, AZMed) which demonstrated high diagnostic accuracy rates, particularly for older children (sensitivity 97.1% versus 91.6% for 5–18-year-olds versus 0–4-year-olds; *p* < 0.001). Whilst other fracture detection products are now commercially available (e.g. BoneView, Gleamer [49]), peer-reviewed publications of such products to date relate only to diagnostic accuracy rates in adults [50] (although paediatric outcomes are available as a conference abstract on the company website [51]).

Most studies in this review specifically chose to develop and apply their AI algorithm for one specific body part, rather than all bones of the paediatric skeleton. Taking the commonest body part for assessment (i.e. the elbow), dedicated algorithms yielded higher diagnostic accuracy rates than the commercially available product for the same body part (which was trained to detect fractures across the entire appendicular skeleton). In this example, the improvement in sensitivity was between 89.5 and 90.7% (for test data, using dedicated algorithms) versus 88% for the generalised tool. Whilst the difference may be small, it could vary across other body parts which we have insufficient dedicated algorithm information for. It will therefore be important to better understand the epidemiology of fractures across different population groups, and whether algorithms that have increased diagnostic accuracies for certain commonly fractured body parts would need to be additionally implemented for certain institutes.

Another aspect highlighted by the present study relates to patient selection, with variable inclusion and exclusion criteria amongst the different studies, a broad range of patient ages (with heterogeneity in bone maturation and mechanisms of injury), with few assessing fractures in children under 2 years (who are more likely to be investigated for suspected physical abuse [52]), or those with inherited bone disorders (e.g. osteogenesis imperfecta). This could be due to fewer children within these categories attending emergency departments to provide the necessary imaging data for training AI models, but the result is that specific paediatric populations may be unintentionally marginalised or poorly served by such new technologies and raises potential ethical considerations about their future usage particularly when performance characteristics are extrapolated beyond the population on which the tool was developed and validated [53]. An example would be an AI tool which could help to evaluate the particular aspects of fractures relating to suspected physical abuse as an adjunct to clinical practice given that many practising paediatric radiologists do not feel appropriately trained or confident in this aspect of imaging assessment [54–57]. Whilst data are limited, one study did address the topic of using AI for identifying suspected physical abuse through the detection of corner metaphyseal fractures (a specific marker of abuse) [58] with a high diagnostic accuracy. Future studies addressing these patient populations, and with details regarding socio-economic backgrounds of cases used for training data, would be helpful to develop more inclusive and clinically relevant tools. Expanding the topic of fracture assessment to address bone healing and post-orthopaedic complications may be another area for further development given that most articles also excluded cases with healing fractures, presence of casts or indwelling orthopaedic hardware.

With the exception of one study, all methods for developing artificial intelligence for fracture detection identified in this review relied on creating or retraining deep convolutional neural networks with the ability to ‘learn’ features within an image to better provide the most accurate desired output classification. Only one study exclusively adopted a more traditional machine learning method using stricter, rule-based computer-aided detection methods for identifying bowing fractures of the forearm [36]. It is unclear whether using a convolutional neural network was unsuitable or less accurate for the detection of these specific fractures or was not attempted due to lack of capability; however, differences in performance of various methods should be compared within the same dataset in relation to not only performance but also resource requirements/costs and other aspects such as ‘exploitability’ of features used by the algorithm. It is likely that the trend for future AI tools for paediatric fracture detection will include development of single or an ensemble of convolutional neural networks to provide optimal performance. Nonetheless, one should not completely disregard simpler machine learning methods, and consider how they can be best employed given the significant computational power and thus carbon footprint produced from training deep learning solutions, especially in the light of current global efforts for creating a more sustainable environment [59].

Although there are fewer publications relating to AI applications for paediatric fractures than in adult imaging, these data have demonstrated that several solutions are being developed and tested with children in mind. Given the current crisis in the paediatric radiology workforce and restricted access to specialist services [60–65], an immediate, accurate fracture reporting service could potentially confer a cost-saving effect [66] and neutralise healthcare inequalities. Nevertheless, there were many limitations to the published literature. For example, health economic analyses and studies assessing whether such algorithms do actually translate into real improvements in patient outcomes are lacking, and it is unclear how generalisable many of the algorithms may be given that most have been tested in a single centre, without external validation and without appropriately powered studies for those that have used multi-reader studies to compare human versus AI performance. Therefore, although this review found that in a subset of the studies the performance of AI algorithms was not significantly different from human performance, this may be due to an under powered sample size. Furthermore, in practice, paediatric radiographs may be interpreted by a range of different healthcare professionals working at different experience levels and with varying subspecialty backgrounds (e.g. general radiologists, paediatric radiologists, musculoskeletal radiologists, paediatricians, orthopaedic surgeons). The current literature only reviews the comparison between AI performance and one kind of healthcare professional. This limits our understanding of who such AI algorithms may best serve and thus how best to implement them.

It should also be recognised that there may be great differences between optimised test performance in validation sets versus the ‘real-world’ impact of implementing such a tool into routine clinical workflows, not only as a consequence of differences/variations in input data, but also usability aspects and pragmatic ability to incorporate such tools into existing workflows. These factors raise questions regarding future widespread implementation and funding of AI solutions as individual hospitals and healthcare systems will require return on their investment at the level of clinical/operational impact rather than pure ‘test performance’[67]. Due to these reasons, it will be necessary for economic analyses and cost and clinical effectiveness studies to be performed to understand whether AI algorithms for fracture detection in children do offer improved benefits.

Improved methods of secure data sharing (possibly with public datasets of paediatric appendicular radiographs) and greater collaboration between hospitals and industrial and academic partners could be beneficial in terms of developing and implementing novel digital tools for paediatric imaging at a lower cost, with future real-world implementation studies. Further research on the topic of AI for paediatric fracture detection should consider aspects that would be helpful to hospital decision-makers, but also consider the uncertainties and bias within test datasets such as the wide age range of patients included, range of different pathologies and injury patterns sustained by children at different stages of maturation which may not all be as accurately evaluated. Improved transparency and subgroup analyses of these, with more robust external validation of emerging and commercially available tools, would provide the necessary evidence for clinicians and hospital managers to better understand whether such technology should be integrated into their own healthcare systems.

There were several limitations to the present study. During the literature review, we included studies that specifically related to paediatric fracture detection. It is possible that some studies may have included children within their population dataset, but did not make this explicit in their abstract or methodology and therefore may have been excluded. Secondly the AI literature is expanding at a rapid rate, and it is likely by the time of publication that newer articles may be available. In order to minimise this effect, an updated review of the literature using the same search strategy was performed immediately before initial article submission and after reviewer resubmission to ensure the timeliness of the findings. We also acknowledge that articles relating to AI applications may be published in open source, but non peer-reviewed research sharing repositories (e.g. arXiv) which were not searched and therefore excluded since only adequately peer-reviewed articles were included. Finally, it proved difficult to consistently extract the required information from the available literature. When assessing for bias, we used a slight adaptation of the QUADAS-2 guideline (whilst future tools are developed [68]) and in some cases the study methodology appeared incomplete or incomprehensible, particularly those written prior to published AI reporting guidelines [69–71]. Accordingly, we included the AI algorithm methodology as an Additional file 1 table due to wide variations in reporting making direct comparisons challenging.

## Conclusions

In conclusion, this review has provided an overview of the current evidence pertaining to AI applications of paediatric appendicular fracture assessment on imaging. There is a wide heterogeneity in the literature with respect to paediatric age ranges, body parts assessed by AI for fracture detection and limited information on algorithm performance on external validation.

Further work is still required, especially for testing solutions across multiple centres to ensure generalisability, and there are currently opportunities for the development of AI solutions in assessing paediatric musculoskeletal trauma across other imaging modalities outside of plain radiography and in certain at risk fracture populations (e.g. metabolic or brittle bone diseases and suspected child abuse cases). Improved research methodology, particularly by using a multicentric dataset for algorithm training with external validation and real-world evaluation, would help to better understand the impact of these tools for paediatric healthcare.

## Supplementary Information

**Additional file 1.** Database search terminologies and details of AI algorithms of included studies.

## Data Availability

All relevant information is provided within the manuscript and Additional file 1. No new data have been generated by this review article.
