# Supplementary material for: Artificial intelligence for radiological paediatric fracture assessment: a systematic review
Source: Insights Imaging. 2022 Jun 3;13:94. doi: 10.1186/s13244-022-01234-3 (PMC9166920; doi:10.1186/s13244-022-01234-3)
Supplement: Supplementary file 1 — Additional file 1. Database search terminologies and details of AI algorithms of included studies. [file 13244_2022_1234_MOESM1_ESM.docx]

**ADDITIONAL FILE 1**

**"Artificial Intelligence for Radiological Paediatric Fracture Assessment: A Systematic Review"**

**Search Terms Used in Database Search**

**Table S1. Medline Search (Limited to 2011 – 2021)**

| **#1** | “imag*”[tw] OR “radiolog*”[tw] OR “radiogra*”[tw] OR "Diagnostic Imaging"[Mesh] OR "Radiology"[Mesh] OR "diagnostic imaging" [Subheading] OR "Radiography"[Mesh] OR "Tomography, X-Ray Computed"[Mesh] | **1,517,882** |
| --- | --- | --- |
| **#2** | “child*”[tw] OR “infant”[tw] OR “toddler”[tw] OR “neonat*”[tw] OR “baby”[tw] OR “teenag*”[tw] OR "Adolescent"[Mesh] OR "Child"[Mesh] OR "Child, Preschool"[Mesh] | **1,409,707** |
| **#3** | "Artificial Intelligence"[Mesh] OR "Deep Learning"[Mesh] OR "Machine Learning"[Mesh] OR "Neural Networks, Computer"[Mesh] OR "Radiographic Image Interpretation, Computer-Assisted"[Mesh] OR "Algorithms"[Mesh] | **210,537** |
| **#4** | “Fractur*”[tw] OR “broken bone*”[tw] OR "Fractures, Bone"[Mesh] | **134,957** |
| **#5** | **#1 AND #2 AND #3 AND #4** | **141** |

**Table S2. Embase Search (Limited to 2011 – 2021)**

| **1** | **Exp fracture/** | **305,140** |
| --- | --- | --- |
| **2** | exp artificial intelligence/ | 55,041 |
| **3** | exp deep learning/ | 20,350 |
| **4** | exp machine learning/ | 282,625 |
| **5** | exp algorithm/ | 464,083 |
| **6** | exp learning algorithm/ | 1,0775 |
| **7** | **2 or 3 or 4 or 5 or 6** | **629,945** |
| **8** | Exp child/ | 2,661,929 |
| **9** | Exp adolescent/ | 1,583,488 |
| **10** | Exp pediatrics/ | 109,839 |
| **11** | Exp infant/ | 990,768 |
| **12** | Exp toddler/ | 5,630 |
| **13** | **8 or 9 or 10 or 11 or 12** | **3,480,136** |
| **14** | Exp radiology/ | 48,783 |
| **15** | Exp imaging/ | 182,206 |
| **16** | Exp computer assisted tomography/ | 1,193,360 |
| **17** | Exp nuclear magnetic resonance imaging/ | 1,077,419 |
| **18** | Exp radiography/ | 1,127,721 |
| **19** | Exp ultrasound/ | 196,254 |
| **20** | Exp nuclear medicine/ | 33,290 |
| **21** | **14 or 15 or 16 or 17 or 18 or 19 or 20** | **2,931,773** |
| **22** | **1 and 7 and 13 and 21** | **132** |

**Table S3. Web of Science (Limited to 2011 – 2021)**

| **#1** | TS=(child) OR TS=(pediatric) OR TS=(infant) OR TS=(neonate) OR TS=(children) OR TS=(adolescent) OR TS=(toddler) | 2,662,936 |
| --- | --- | --- |
| **#2** | TS=(Deep AND learning) OR TS=(DL) OR TS =(Hierarchical AND Learning) OR TS=(deep AND learning AND algorithm) OR TS=(Artificial AND intelligence) OR TS=(Neural AND Networks And Computer) OR TS=(machine AND learning) OR TS=(computer AND assisted AND detection) | 980202 |
| **#3** | TS=(fractur* AND bone) OR TS=(fracture) OR TS=(broken AND bone) | 604,575 |
| **#4** | TS=(Tomography AND X-Ray AND Computed) OR TS=(Multidetector AND Computed AND Tomography) OR TS=(Computed AND Tomography) OR TS=(CT) OR TS=(CAT AND scan) OR TS=(magnetic AND resonance AND imaging) OR TS =(MRI) OR TS=(magnetic AND resonance) OR TS=(MRI AND scan) OR TS=(ultras*) OR TS =(US) OR TS=(US AND Scan) OR TS=(radiolog*) OR TS=(imag*) OR TS =(radiograph*) OR TS=(X-ray) OR TS=(radiology) OR TS=(diagnostic AND imaging) OR TS=(nuclear AND medicine) OR TS=(PET) OR TS=(positron AND emission AND tomography) | 8,584,399 |
| **#5** | **#1 AND #2 AND #3 AND #4** | **126** |

**Table S4. Cochrane Library (Limited to 2011 – 2021)**

| **#1** | Child:ti,ab,kw (word variations have been searched) | |
| --- | --- | --- |
| **#2** | Artificial intelligence:ti,ab,kw (word variations have been searched) | |
| **#3** | Fracture:ti,ab,kw (word variations have been searched) | |
| **#4** | Imaging:ti,ab,kw (word variations have been searched) | |
| **#5** | **#1 AND #2 AND #3 AND #4** | **0** |

**Table S5: Details of artificial intelligence algorithm developed or used, organised by publication date**

*DCNN – deep convolutional neural network; ANN – artificial neural network; CADx – Computer Aided Detection for Diagnosis*

| **Author, year** | **Model Type** | **Algorithm Development Details** |
| --- | --- | --- |
| **Zhou, 2016** | CADx | Computer aided detection tool was designed to detect bowing fractures of the radius/ulna.  Initially the degree of bowing of normal bones was established by creating a semi-automated bone extraction method based on radius curvature. An analytical equation was developed to determine the maximal bending allowed within the bone before a ‘buckle/bowing’ fracture is determined.  For the bone extraction process, a crop tool extracted the radius or ulna from the image and the co-ordinates for the outer boundary of the bones was fitted through edge detection. The co-ordinates were fitted to a function y=f(x), where f is a 6^th^ degree polynomial. The radius of the curvature for each point/co-ordinate on the boundary of the bone was plotted and the co-ordinates for the maximum curvature values were taken as regions for bone extraction. The extracted bone boundary was then used for analysis by calculating the central angle (angle by the arc that covers both ends of the bone) and circle curvature (reciprocal of radius curvature). Threshold value for optimal parameters of central angle/circle curvature to determine presence of a bowing fracture was determined using independent two-sample t-test for each group.  A graphical user interface (GUI) to automatically perform image pre-processing, extraction, data analysis was created and built into a standalone application for clinical usage. |
| **England, 2018** | DCNN  Architecture not mentioned | Models were trained and tested using Keras (version 2.1.3), run on top of TensorFlow (version 1.5.0, Google Brain Team) on a computer with a 3.7-GHz processor (Core i7–8700 K, Intel), 16- GB random access memory, and an 11-GB graphics card (GTX 1080 Ti, Nvidia).  Hyperparameters were systematically optimized by training 465 models with different hyperparameter settings and assessing performance on the validation set. For each of the 465 trials, hyperparameters were chosen randomly from a distribution of interest. Initial learning rate and dropout rate were selected from uniform log-domains in early trials and were later selected from narrower uniform linear domains. Other continuous hyperparameters were selected from uniform linear domains. Integer hyperparameters were selected from uniform linear domains of integers. Boolean hyperparameters were selected from binary (true or false) domains.  For each trained model produced during the hyperparameter search, the ROC curve was plotted by systematically varying the threshold (i.e., the cutoff point between 0 and 1 above which the model output is considered diagnostic of elbow effusion) and calculating true-positive rate and false-positive rate. The ROC AUC was calculated using scikitlearn (version 0.19.1), an open-source machine learning library, and the model with the highest ROC AUC on the validation set was selected as the final model.  Ninety-five percent CIs were calculated by use of the DeLong method using the pROC package.  Optimum operating threshold for calculation of sensitivity, specificity, and accuracy was chosen as the cut-off point that maximizes the Youden index (defined as sensitivity + specificity − 1) on the validation set. |
| **Malek, 2016** | DCNN (Architecture not mentioned) and SOM | Both supervised and unsupervised artificial neural networks (ANN) were applied using Neural Network Toolbox in MATLAB. Back-propagation learning with one hidden layer was employed for the supervised ANN part. The ANN model was constructed with three main layers, where input layer included 7 nodes, hidden layers included 10 nodes, and output layers included 1 node. The network was trained with scaled conjugate gradient.  Three data sets are used for the ANN model development: a training set, a test set, and a validation set. Backward elimination method was used to eliminate insignificant variables. Elimination process involved starting with all candidate variables, testing the deletion of each variable using a chosen model comparison criterion, deleting the variable (if any) that improved the model the most by being deleted, and repeating this process until no further improvement was possible.  The inputs were ranked using Pearson correlation coefficient before carrying out backward elimination process. Root mean square error (RMSE), receiving operator characteristic (ROC), area under the curve (AUC) and accuracy rate were the main assessment criteria adopted to evaluate results.  Self-Organizing Maps (SOM) were also used in this study to ordinate fracture input variables with respect to healing time.  As a result of the training of the unsupervised ANN, the Euclidian distance between the inputs are calculated and visualized as distance matrix (U-matrix). SOM reduces data dimensions by producing a map of 1 or 2 dimensions which plot the similarities of the data by grouping similar data items together. Thus, SOM reduce dimensions and display similarities. This enables the discovery or identification of features or patterns of most relevance through data reduction and projection. The u-matrix representation of SOM visualizes the distances between neurons. The distance between the adjacent neurons is calculated and presented with different colourings between the adjacent nodes. |
| **Rayan, 2019** | DCNN  Xception architecture | Training of the DCNN was performed in two phases.  For phase 1, as the image classifier, the Xception architecture was modified to accept single-channel grey-scale input.  This model was trained without pretrained weights. This is referred to as the ‘vision model’. The input dimensions of the vision model were modified to be 500 × 500 × 1 (as represented by single-channel floating point values in the range of 0 to 1).  After the final feature extraction layer with adaptive global average pooling, where high-level image features are extracted upon forward pass through the model, a two unit dense layer was added, following a scaled exponential linear units activation function. The model specifically optimized a cross entropy loss with the AMSGrad variant of the Adam optimizer.  The model was trained for 100 epochs, with the best model from the entire training run chosen to be used in phase 2.  The lowest validation loss occurred at epoch 83. The learning rate of the optimizer was initialized to 0.001 and was reduced by 90% if the loss did not reduce over the course of 10 epochs to a minimum of 0.00001 (two reductions in total).  For the second phase of training, the vision model was used as a high-level feature extractor within a larger model encompassing a recurrent neural network. Parameters for the trained CNN were locked during this phase of training. A 512-unit dense fully connected layer served as the bridge between the CNN and the 512-unit gated recurrent unit (GRU). The GRU was followed by a two-unit dense layer that output floating point values. This model processed three images in series and made its decision only after viewing all three images.  The output values of the model were interpreted after passing it through a softmax layer by examining the scores in the positive category. A simple scoring system was used where values in the range of 0.0 to 0.5 were defined as normal, and values in the range of 0.5 to 1.0 were defined as abnormal. Values in the range of 0.25 to 0.75 were considered low confidence, and those outside this range as high confidence. |
| **Choi, 2020** | DCNN  ResNet-50 architecture | The deep-learning algorithm was developed using Keras (version 2.1.5, https://keras.io/), a Python-based high-level deep-learning library, run on top of Tensorflow (version 1.4.0; Google Brain Team, Mountain View, CA), on a computer with an Intel Core i7-7500U 2.7-GHz processor (Intel, Santa Clara, CA), a 16-GB RAM, and an NVIDIA GeForce 940MX GPU (NVIDIA, Santa Clara, CA).  An image classification model was defined that took 2 images with the same size of 200 x 200 as input. Each image was input simultaneously to 2 identical ResNet-50 models without the top classification layer pretrained on the ImageNet dataset.  Output features of each submodel were concatenated and passed into a fully connected layer and a Softmax activation function that produced prediction values between 0 and 1 for the 2 labels. The concept of combining identical submodels in parallel was adopted from the Siamese neural network which has been introduced in several previous studies that use a multiview neural network architecture in mammography.  The hyperparameters used for training were as follows: categorical cross-entropy as the loss function; stochastic gradient descent optimizer with a learning rate of 1 × 10^−3^ a decay of 1 × 10^−6^, a momentum of 0.9, and Nesterov momentum; 31,625 iterations; and a batch size of 4.  Source code for deep-learning algorithm is available at <https://github.com/jwc-rad/elbowfx>. |
| **Starosolski, 2020** | DCNN  Xception architecture | Modifications were made to the Xception network: 2 modules of fully convoluted reasoning layers with 5x128 nodes each, and incorporating drop-out segments placed on top of the existing network.  The Xception architecture consists of 36 convolutional layers structured into 14 modules, creating a feature recognition foundation of the network. In this study, the weights of the first 9 modules were conserved, allowing for training of the next 5 Xception modules plus 5 reasoning additional modules not included in the original Xception network.  The training was done in two phases. In phase 1, a short training set preliminary weights in the top 2 reasoning layers over 10 epochs was used. In phase 2, the weights of pretrained 5 layers of Xception were adjusted and the 2 additional reasoning layers were built on the top of the existing network to fine-tune the best accuracy in the validation set. Phase 2 was done over 300 epochs with the Nesterov Adam optimizer (NADAM). The final training period took ~230 minutes.  Network implementation and training were conducted in the Keras 2.1.4 environment with Python 2.7.12, and Tensorflow 1.3.0, on one Nvidia Tesla K80 GPU, on a dual Xeon CPU workstation. |
| **Dupuis, 2021** | DCNN  Commercially available product, based on RetinaNet architecture | A commercially available product was used (Rayvolve®, AZMed)  The Rayvolve algorithm is an ensembling algorithm composed of five object detection models based on RetinaNet architecture. The object detection algorithms are mostly composed of trainable convolutional layers, non-trainable max-pooling layers and trainable other layers. Each object detection model outputs a list of bounding boxes (this list can be empty if no fracture is detected by the object detection model) with their respective confidence scores.  In choosing the final list of bounding boxes, a majority vote between the predicted bounding boxes of each model is selected. To be kept, a bounding box would have a non-null intersection with at least two others bounding boxes from two different object detection models. The list of bounding boxes outputted by the ensembling algorithm are the bounding boxes that have been outputted by at least three object detection model and thus, an overlapping between three bounding boxes is necessary to keep one final bounding box.  Between the overlapping bounding boxes, the one with the highest score was kept. Each object detection model has the same right of vote. During the ensembling, a final step is done to filter the bounding boxes with a score lower than a determined threshold. |
| **Zhang, 2021** | DCNN  Architecture not mentioned | Using a brute force approach, several models were trained with varying numbers of convolutional layers and fully connected layers. Three networks that gave the highest accuracy were selected to be part of the ensemble. The output of each ensemble represented the median prediction of the three CNNs included. Separate ensembles were trained for volar sagittal and dorsal sagittal scans within the dataset.  The ranges of various network parameters used:  Number of convolutional layers: 2-5, step size = 1  Number of fully connected layers: 1-2, step size = 1  Optimizers: Stochastic Gradient Descent (SGD), RMSpropr, Adagrad, Adadelta, Adamaz ADAM.  Drop out: 0-50%, step size = 10  Loss Function: Cross-entropy (CE)  Epochs: 80 |
| **Tsai, 2022** | DCNN  ResNet-34 architecture | The model used the cross-entropy loss function within the ResNet-34 model for binary classification. A class weighting scheme was incorporated into the loss function to handle class imbalance in the training dataset. In particular the samples were weighted as the inverse of the class frequencies. For network hyperparameters, the number o classes was set at 2, and empirically chosen to have a larning rate of 0.00005, 250 epochs and a batch size of 5. An Adam optimizer inspired by two popular gradient descent methods (AdaGrad and RMSProp algorithms) was used to minimize the cross-entropy loss function and train the residual neural network.  The proposed model was implemented in PyTorch version 1.6.0. The residual neural network model was trained and tested on an Enkefalos 2.0 cluster (28 nodes with 750 CPU cores, 13 GPUs, and 3.5 TB memory) built on Intel architecture with a CentOS 7.6 Linux operating system. This particular job ran on a NVIDIA T4 GPU with 16 GB memory. |
